# 

**Published:** 1893-08

**Authors:** 


### *MAGAZINES RECEIVED.*

### LIPPINCOTT'S MAGAZINE FOR AUGUST, 1893.

The complete novel in the August number of *Lippincott's* is "In the Midst of Alarms," by Robert Barr (Luke Sharp). It is a tale of the Fenian invasion of Canada in 1871.

The sixth in the series of *Lippincott's* Notable Stories is "Jane's Holiday," by Valerie Hays Berry. It is illustrated.

In "The Lady of the Lake" Julian Hawthorne describes some of the statuary and other attractions of the Columbian Exposition.

The Athletic Series is continued in an article on "The National Game," by Norton B. Young. It is accompanied by portraits of several leading players.

"Zachary Taylor, his Home and Family," is by the President's grandniece, Mrs. Annah Robinson Watson. It corrects certain popular errors (as that concerning the first marriage of Jefferson Davis), and gives much interesting information about one of the least known of our great men. This article is illustrated, as is another valuable biographical paper, "A Philadelphia Sculptor" (William Rush), by E. Leslie Gilliams.

W. H. Babcock discusses "Supermundane Fiction," and M. Crofton, in "Men of the Day," presents brief sketches of Sir J. E. Millais, Sir Arthur Sullivan, General Diaz and Philip D. Armour.

The poetry of the number is by Clara Jessup Moore, Howard Hall and M. H. G.

---

The midsummer *Cosmopolitan*, the first at the new price of twelve and a half cents per copy, though unchanged in size, excels any other issue of that mazazine in the number of its distinguished contributors, in the interest of its contents and in its overflowing illustrations by famous artists. Fançois Coppée, William Dean Howells, Camille Flammarion, Andrew Lang, Frank Dempster Sherman, H. H. Boyesen, Charles DeKay, Thomas A. Janvier, Colonel Tillman, Agnes Repplier and Gilbert Parker are a few of the names which appear on its title page. Three frontispieces, all by famous artists, furnish an unusual feature, and among the artists who contribute to the 119 illustrations adorning its pages, are Laurens, Reinhart, Fenn, Toussaint, Stevens, Saunier, Fitler, Meaulle and Franzen. The midsummer number is intended to set the pace for the magazine at its new price of twelve and a half cents a copy, or $1.50 a year. The magazine remains unchanged in size and each issue will be an advance upon its predecessors. Literally, every known country is being ransacked for material in the hope to bring *The Cosmopolitan* forward as the leading magazine in the world.

